# Peripheral T-Cell Lymphomas: Therapeutic Approaches

**DOI:** 10.3390/cancers14092332

**Published:** 2022-05-08

**Authors:** David Sibon

**Affiliations:** 1 Lymphoid Malignancies Department, Henri Mondor University Hospital, AP-HP, 94000 Créteil, France; david.sibon@aphp.fr; 2 Faculty of Medicine and Health, Campus Henri Mondor, Paris-Est Créteil University, 94000 Créteil, France

**Keywords:** PTCL, CHOP, CHOEP, BV-CHP, hematopoietic stem-cell transplantation, targeted treatments

## Abstract

**Simple Summary:**

Peripheral T-cell lymphomas are a group of rare cancers of T cells or natural killer cells, most often with a poor prognosis. In recent years, significant progress has been made through the development of more specific therapies. This review aims to provide an up-to-date overview of current treatments in nodal PTCL.

**Abstract:**

Peripheral T-cell lymphomas (PTCLs) are a heterogeneous group of rare neoplasms of mature T cells or natural killer (NK) cell. PTCLs usually have an aggressive course and a poor outcome. In recent years, significant progress has been made in the knowledge of the molecular lymphomagenesis of PTCLs, and through the development of new, more specific therapeutic molecules, one can hope in the coming years for more personalized medicine and improved patient prognosis. This review aims to provide an up-to-date overview of the current therapeutic approaches in nodal PTCLs.

## 1. Introduction

**Figure 1:**
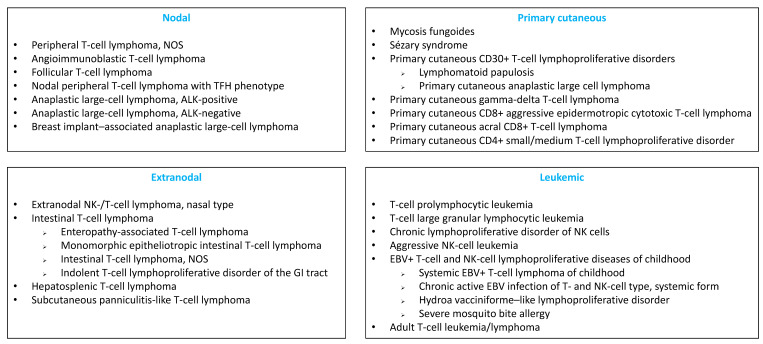
Classification of mature T- and NK-cell neoplasms according to the 2017 WHO classification.

Peripheral T-cell lymphomas (PTCLs) are neoplasms of mature T cells or natural killer (NK) cells. PTCLs are a heterogeneous group of rare lymphoid malignancies, comprising 31 entities in the updated 2017 World Health Organization (WHO) classification [1]), which can be classified into 4 groups: nodal, extranodal, leukemic and primary cutaneous (Figure 1). PTCLs account for only 5–10% of all noncutaneous lymphomas, with geographic and ethnic variations [2,3,4]. The pathogenesis and the mechanisms of PTCL transformation are complex and have begun to be deciphered in recent years [5].

PTCLs usually have an aggressive course and a poor outcome. The rarity and heterogeneity of these diseases limit the study of new treatments in clinical trials. Until recent years, few therapies have been developed specifically for PTCLs. The situation has now changed, with many new drugs, but too few patients available to test them. Over recent years, our understanding of PTCLs molecular pathogenesis has advanced remarkably, and we can expect the development of specific treatments for individualized PTCL entities, or the targeting of a specific protein or biological pathway shared by certain entities. This review provides an update on therapies for nodal PTCLs.

## 2. Frontline Treatment of PTCLs

Given the rarity of PTCLs, there is a lack of randomized studies, and most therapeutic approach paradigms for PTCLs have been derived from treatments developed for aggressive B-cell non-Hodgkin lymphomas (NHLs). As a result, a cyclophosphamide, doxorubicin, vincristine, and prednisone (CHOP) regimen was considered the standard therapy despite results showing that it was suboptimal (except in ALK+ anaplastic large cell lymphoma (ALCL)). Thus, for the main nodal PTCLs (AITL, PTCL-NOS, and ALK− ALCL), the 2- and 5-year progression-free survival (PFS) is approximately 35% and 25%, and the 2- and 5-year overall survival (OS) is 45% and 35%, respectively [6,7,8,9,10,11,12,13].

### 2.1. How to Improve CHOP?

For more than 20 years, several studies have attempted to improve the results of CHOP in PTCLs, but very few have succeeded. Here, we present these studies as well as the unresolved questions.

#### 2.1.1. CHOEP and Other Intensive Chemotherapy Regimens

Adding etoposide to CHOP (CHOEP) was one of the first attempts to improve PTCL prognosis. Although there have been no randomized studies comparing CHOP and CHOEP regimens in PTCLs, a number of retrospective or phase 2 prospective studies have suggested a benefit of CHOEP. Due to the increased toxicity of CHOEP, this regimen is usually preferred in patients less than 60–65 years old. Table 1 summarizes the main studies that evaluated CHOEP in PTCLs [7,9,12,14,15,16,17,18,19]. Apart from a Korean retrospective study which did not find any benefit of CHOEP over CHOP, suggesting that Asian patients would not benefit from the addition of etoposide, most studies suggested an advantage of CHOEP over CHOP in PFS and/or OS. This benefit is most evident in patients with ALK+ ALCL, with two retrospective studies dedicated to this entity showing a benefit in PFS and OS, independent of the international prognostic index (IPI) [12,19]. In the absence of a randomized study, the real benefit of using CHOEP in PTCLs is still a matter of debate.

Besides the CHOEP regimen, some intensive polychemotherapies have been studied, such as dose-adjusted etoposide, prednisone, vincristine, cyclophosphamide, and doxorubicin (DA-EPOCH) [20,21], Mega-CHOEP [22], and cyclophosphamide, vincristine, doxorubicin, dexamethasone/methotrexate, cytarabine (HyperCVAD) [23,24]. A total of 2 phase 2 studies reported the results of DA-EPOCH in PTCLs. The first 1 enrolled 24 patients with systemic ALCL (ALK+, *n* = 15; ALK−, *n* = 9; median age 38 years), and reported, after a median follow-up of 14.4 years, event-free survival (EFS) probabilities of 72% and 62.5%, and OS probabilities of 78% and 87.5%, in ALK+ and ALK− ALCL patients, respectively [20]. In the Japanese study, 41 patients (mostly PTCL-NOS and AITL) were treated with DA-EPOCH. Their 2-year PFS and OS were 53% and 73%, respectively [21]. The German High-Grade Non-Hodgkin Lymphoma Study Group (DSHNHL) reported the results of MegaCHOEP in 33 PTCL patients (no ALK+ ALCL) [22]. A total of 22 out of 33 patients (66.7%) received all of the planned therapy. The main reasons for the early discontinuation of treatment were progressive or stable disease in 4 patients (12%), and extensive toxicity in 6 patients (18%), with 2 therapy-related deaths. The 3-year EFS and OS were 26% and 45%, respectively. The Australasian Leukaemia and Lymphoma Group reported a phase 2 study of a modified hyperCVAD frontline therapy in 26 PTCL patients [23]. This regimen resulted in similar outcomes to CHOP-like chemotherapy and was associated with significant toxicity. Similar results were reported by the MD Anderson Cancer Center in a retrospective study [24]. Overall, these intensive regimens do not seem to provide any benefit compared to CHOP/CHOEP, and their toxicity is often more marked.

#### 2.1.2. Combination of CHOP with Novel Agents

With the development of new drugs, a number of prospective phase 1 and 2 studies have evaluated CHOP associated with monoclonal antibodies, small molecule inhibitors, epigenetic modifiers, or antimetabotite [25,26,27,28,29,30,31,32,33,34]. Table 2 summarizes these studies. Only 3 of these combinations (brentuximab vedotin (BV) + cyclophosphamide, doxorubicin, and prednisone (CHP), alemtuzumab-CHOP, and romidepsin-CHOP) were subsequently evaluated in a randomized, phase 3 trial versus CHOP. A phase 2 study was recently presented at the 2021 ASH meeting, assessing the addition of etoposide to BV-CHP (BV-CHEP regimen) in adults with newly diagnosed PTCL (including ALK+ ALCL with IPI ≥ 2) and CD30 expression ≥ 1% on tumor cells by immunohistochemistry [35]. Patients received BV consolidation and autoSCT was allowed. The primary endpoint was the CR rate after BV-CHEP. Overall, 48 patients were enrolled (including 3 ALK+ ALCL). The CR rate was 80% (ORR 91%), and the treatment was tolerable. The BV-CHEP regimen could be of special interest in patients with ALK+ ALCL, in whom etoposide and BV have demonstrated a benefit.

#### 2.1.3. Randomized Studies Challenging CHOP

Very few randomized studies have tried to challenge CHOP in PTCLs. Three of them combined CH(O)P with a novel agent, and two others each evaluated a new chemotherapy regimen (etoposide, ifosfamide, cisplatin, doxorubicin, bleomycin, vinblastine and dacarbazine (VIP-reinforced-ABVD); gemcitabine, cisplatin, and methylprednisolone (GEM-P)) [11,13,36,37,38,39]. Only ECHELON-2 study assessing BV-CHP reached its primary endpoint. Table 3 summarizes the main results of the 5 studies.

LTP-95 is a randomized, phase 3 study that compared the etoposide, ifosfamide, cisplatin alternating with doxorubicin, bleomycin, vinblastine, dacarbazine (VIP-reinforced-ABVD; VIP-rABVD) regimen to CHOP as frontline treatment in PTCLs. The primary objective was the 2-year EFS (events were cessation of treatment for toxicity or progression, relapse, or death from any cause). A total of 88 patients were randomized (43 to VIP-rABVD and 45 to CHOP), including 10 with ALK+ ALCL. After a median follow-up of 9 years, the 2-year EFS was 45% with VIP-rABVD and 41% with CHOP, which was not statistically different.

CHEMO-T is a randomized, phase 2 study that compared the gemcitabine, cisplatin, and methylprednisolone (GEM-P) regimen with CHOP in PTCL patients ≥ 18 years [11]. ALK+ ALCL were not included. The primary endpoint was complete response (CR)/CR unconfirmed (CRu) rates on completion of chemotherapy. After the inclusion of 87 patients (44 to GEM-P and 43 to CHOP), a planned review of efficacy data by the independent data monitoring committee (IDMC) showed that fewer patients had achieved a CR/CRu with GEM-P (46%) than with CHOP (62%). The IDMC concluded there was a high likelihood that GEM-P would be inferior to CHOP at the end of the trial, and the trial was closed early.

ECHELON-2 is a randomized, phase 3 study comparing BV-CHP with CHOP in patients ≥ 18 years with CD30+ PTCLs [37]. Enrollment targeted 75% of patients with systemic ALCL (sALCL) to ensure the secondary endpoint of PFS in this subgroup could be appropriately assessed. Key inclusion criteria were as follows: age ≥ 18 years, eligible histology according to WHO 2008 classification (ALK+ ALCL with IPI ≥ 2, ALK− ALCL, PTCL-not otherwise specified (PTCL-NOS), angioimmunoblastic T-cell lymphoma (AITL), adult T-cell leukemia/lymphoma (ATLL), enteropathy-associated T-cell lymphoma (EATL), or hepatosplenic T-cell lymphoma (HSTL)), performance status (PS) 0–2; CD30 should be ≥10% of neoplastic cells (in cases where enumeration of neoplastic cells was not possible, total lymphocytes may have been used) by local review. Primary endpoint was modified PFS by independent central review, the events of which were relapse/progression, death from any cause, or receipt of subsequent systemic chemotherapy for failure of frontline treatment. Randomization was stratified by histological subtype according to local pathology assessment (ALK+ ALCL vs. all other histologies) and IPI score (0–1 vs. 2–3 vs. 4–5). Patients received 6 or 8 cycles (at the investigator’s discretion at registration) of either BV-CHP or CHOP. Consolidative stem-cell transplantation or radiotherapy after treatment was permitted at the investigator’s discretion (stem-cell transplantation intent was prespecified before the first cycle of chemotherapy). In all, 226 patients were assigned to each arm. Median PFS was 48.2 months in the BV-CHP arm vs. 20.8 months in the CHOP arm (hazard ratio (HR) 0.71 (95% confidence interval (CI) 0.54–0.93), *p* = 0.01), with 3-year PFS of 57% vs. 44%. OS was also significantly improved in the BV-CHP arm, with 3-year OS of 77% vs. 69% (HR 0.66 (95% CI 0.46–0.95), *p* = 0.02). Incidence and severity of febrile neutropenia and peripheral neuropathy were similar between arms. Based on ECHELON-2, the Food and Drug Administration approved brentuximab vedotin for the treatment of adult patients with previously untreated sALCL or other CD30-expressing PTCLs, including AITL and PTCL-NOS, in combination with cyclophosphamide, doxorubicin, and prednisone. The European Medicines Agency (EMA)’s approval was more restricted, brentuximab vedotin being indicated in combination with cyclophosphamide, doxorubicin, and prednisone for adult patients with previously untreated sALCL. The EMA’s decision was based on the fact that the subgroup analysis of AITL (*n* = 54) and PTCL-NOS (*n* = 72) showed no benefit of BV-CHP in PFS and OS compared to CHOP. Although the study was not powered to demonstrate a benefit in these subgroups, the observed confidence interval makes the existence of a clinically relevant difference between BV-CHP and CHOP unlikely in AITL and PTCL-NOS.

An updated analysis was recently published after prolonged follow-up [38]. The estimated 5-year PFS was 51% for the BV-CHP arm versus 43% for the CHOP arm (*p* = 0.0077; HR 0.70 (0.53–0.91)), and the estimated 5-year OS was 70% for the BV-CHP arm versus 61% for the CHOP arm (*p* = 0.0424; HR 0.72 (0.53–0.99)). In subgroup analyses, the PFS improvement was significant only for sALCL (by pooling ALK+ and ALK− sALCL, but not in each individual entity).

Thus, ECHELON-2 mainly demonstrated a benefit of BV-CHP on CHOP in ALCL. However, some questions remain unanswered. The choice of CHOP as the control arm rather than CHOEP could be questioned. Indeed, as indicated previously, two retrospective studies have shown a benefit of CHOEP over CHOP in PFS and OS in ALK+ ALCL [12,19], and the question remains open for ALK− ALCL. Furthermore, since ALK+ ALCL IPI 0–1 were not included in ECHELON-2, we support the claim that CHOEP should be the preferred option for these patients, given the lack of data for BV-CHP.

Another point of discussion concerns the rearrangement of *DUSP22* (*DUSP22*-R), which is present in 20 to 30% of all ALK− ALCL. Until recently, the prognostic impact of *DUSP22*-R was uncertain, with 5-year OS ranging from 40% to 90% in the 2 main studies, including 12 and 22 patients, respectively [40,41]. Recently, a LYSA and TENOMIC study compared the outcome of 45 ALK− ALCL patients with *DUSP22*-R and 55 non-rearranged (*DUSP22* non-R) cases [42]. The 5-year PFS was 46% and 21% for the *DUSP22*-R and non-R patients, respectively (*p* < 0.01), and 5-year OS was 57% and 45% for the *DUSP22*-R and non-R patients, respectively (*p* = 0.32). Since ECHELON-2 did not analyze *DUSP22*-R, it is not known whether the distribution of *DUSP22*-R was balanced between BV-CHP and CHOP arms, and to what extent this could have impacted the PFS difference observed between BV-CHP and CHOP arms in patients with ALK− ALCL.

The last questionable point concerns the correlation between the CD30 expression and the efficacy of BV in non-ALCL subtypes (ALCL tumor cells are always positive for CD30 by definition). In ECHELON-2 inclusion criteria, by local review, CD30 should be ≥10% of neoplastic cells, but in cases where enumeration of neoplastic cells was not possible, total lymphocytes may have been used. This means that CD30 expression by non-tumor cells could be used (such cells can be seen in PTCLs, notably in AITL), which complicates the evaluation of the correlation between expression and efficacy. Moreover, after central review, some patients had <10% CD30+, and even no expression. Finally, a study of CD30 expression above vs. below median (or at 10%) did not predict response to BV-CHP in ECHELON-2 non-ALCL subtypes, as responses were seen across CD30 levels [43].

In summary, ECHELON-2 is an important study in the field of PTCLs. As the study did not report survival curves for each PTCL entity, comparison with other studies is difficult. Questions regarding the comparison between BV-CHP and CHOEP, especially in ALK+ ALCL, the impact of *DUSP22*-R in ALK− ALCL, and the optimal CD30 expression level need to be clarified.

ACT-2 is a randomized, phase 3 study that compared alemtuzumab-CHOP (A-CHOP) to CHOP in 116 PTCL patients (58 in each arm), aged 61–80 years [39]. ALK+ ALCL were not included. The primary endpoint was 3-year EFS. CR/CRu rates were 60% and 43% for A-CHOP and CHOP, respectively. The 3-year EFS, PFS, and OS were 27%, 28%, and 37% for A-CHOP, vs. 24%, 29%, and 56% for CHOP, respectively, with no significant differences. A-CHOP increased hematotoxicity, resulting in more grade ≥3 infections (40% vs. 21%) and death due to infections (4 vs. 1) than CHOP. In summary, A-CHOP increased CR rates, but it did not improve survival due to toxicity.

The Ro-CHOP randomized, phase 3 study compared romidepsin-CHOP vs. CHOP in PTCL patients aged 18–80 years [13]. ALK+ ALCL were not included. The primary end point was PFS. For the 421 enrolled patients (Ro-CHOP, *n* = 211; CHOP, *n* = 210), median PFS was 12.0 vs. 10.2 months, and 2-year PFS was 43% vs. 36% for Ro-CHOP vs. CHOP (*p* = 0.096), respectively. The 2-year OS was 64% vs. 63% for Ro-CHOP vs. CHOP (*p* = 0.48), respectively. Preplanned PFS analyses were conducted in subgroups with potential prognostic/predictive factors for the intent-to-treat population. There was no statistically significant difference in PFS between the Ro-CHOP and CHOP arms for any subgroup analyzed. Exploratory analysis in centrally-confirmed AITL and other nodal lymphomas of T follicular helper (TFH) cell origin showed prolonged PFS in the Ro-CHOP arm compared to the CHOP arm, with a median PFS of 19.5 months vs. 10.6 months ((HR 0.69 (95% CI 0.48–1.00; *p* = 0.046)), respectively. Grade 3/4 cytopenias were more frequent in the Ro-CHOP arm, with thrombocytopenia (50% vs. 10%), neutropenia (49% vs. 33%), and anemia (47% vs. 17%). In summary, the addition of romidepsin to CHOP did not improve PFS nor OS, and increased toxicity. The suggested benefit in TFH-like lymphomas (AITL and TFH PTCL) warrants further investigation.

### 2.2. The role of Consolidative Hematopoietic Stem-Cell Transplantation

#### 2.2.1. Autologous Stem-Cell Transplantation

##### Prospective Studies

A total of 5 prospective phase 2 studies, one of which was subsequently updated, assessed upfront autologous stem-cell transplantation (autoSCT) in PTCLs [14,44,45,46,47,48]. Most of these studies excluded ALK+ ALCL, which has a better prognosis. Table 4 summarizes the main results of these studies.

The largest of these trials, the NLG-T-01 study, was conducted by the Nordic Lymphoma Group [14], and was the basis for the current European guidelines [49]. Overall, 160 patients with histopathologically confirmed PTCL (excluding ALK+ ALCL) were treated with 6 cycles of CHOEP-14 (etoposide was omitted in patients > 60 years). Patients in CR or partial response (PR) proceeded to consolidation with a carmustine, etoposide, cytarabine, and melphalan (BEAM) conditioning regimen, followed by autoSCT. A total of 115 patients (72%) underwent autoSCT. Treatment-related mortality was 4%. After a median follow-up of 60.5 months, 5-year PFS and OS rates were 44% and 51%, respectively. Subtype-specific analysis revealed the highest PFS and OS occurring in patients with ALK− ALCL (5-year PFS and OS of 61% and 70%, respectively). Patients with AITL had 5-year PFS and OS of 49% and 52%, respectively; patients with PTCL-NOS had 5-year PFS and OS of 38% and 47%, respectively; and patients with EATL had 5-year PFS and OS of 38% and 48%, respectively.

The COMPLETE prospective, multicenter cohort study reported the outcome of 119 patients with nodal PTCL in CR1 [50]. Among them, 36 patients underwent autoSCT in CR1. In multivariate analysis, autoSCT was independently associated with improved OS (HR, 0.37; 95% CI, 0.15–0.89).

##### Retrospective Studies

The largest retrospective studies, which included at least 100 patients, are presented in Table 5 with their main results [9,12,51].

#### 2.2.2. Allogeneic Stem-Cell Transplantation

The majority of allogeneic stem-cell transplantation (alloSCT) studies in PTCLs concern relapsed or refractory patients, and few frontline data are available. Here we present the most relevant frontline studies.

##### Prospective Studies

In 2014, the FIL group reported the results of a phase 2 study assessing intensified chemo-immunotherapy, with or without stem-cell transplantation, in newly diagnosed patients with PTCL [52]. On the basis of donor availability, patients ≤ 60 years old in response received alloSCT or autoSCT. Among 61 patients ≤ 60 years old, 38 (62%) responded and received alloSCT (*n* = 23) or autoSCT (*n* = 14), and one patient in CR was not transplanted. After a median follow-up of 40 months, the 4-year OS, PFS and disease-free survival (DFS) rates were 49%, 44%, and 65%, respectively. The study was not powered to evaluate the differences between autoSCT and alloSCT.

The randomized AATT phase 3 trial was recently published [17]. This study is the only phase 3 study assessing a transplant strategy in the frontline treatment of PTCLs. It was hypothesized that alloSCT could improve outcomes over autoSCT. Key inclusion criteria were as follows: age 18–60, stage II-IV or age-adjusted IPI (aaIPI) > 0, PS 0–3, and a nodal or extranodal PTCL excluding ALK+ ALCL. Patients were randomized upfront to receive 4 cycles of CHOEP-14, 1 cycle of DHAP (dexamethasone, cytarabine, and cisplatin or carboplatin), and autoSCT or alloSCT. In the absence of progressive disease (PD) at the time of restaging, patients continued in the study and were scheduled to receive either BEAM high-dose chemotherapy followed by autoSCT or myeloablative conditioning with fludarabine, busulfan, and cyclophosphamide, followed by alloSCT. The primary end point was EFS at 3 years. Overall, 104 patients were included. The data safety and monitoring board, in agreement with the study steering committee, stopped randomization and recruitment because a planned interim analysis had shown that the study was highly unlikely to meet the primary end point. The transplant-related mortality rate (TRM) contributed to this decision. After a median follow-up of 42 months, the 3-year EFS after alloSCT was 43% vs. 38% after autoSCT. The 3-year OS was 57% vs. 70% after alloSCT or autoSCT, respectively, without significant differences between treatment arms. Interestingly, there was no relapse among the 21 responding patients who underwent alloSCT, as opposed to 13 of 36 patients (36%) proceeding to autoSCT. However, 8 of the 26 patients (31%) died of transplant-related toxicity after alloSCT vs. none of the 41 patients after autoSCT.

##### Retrospective Studies

The Société Francophone de Greffe de Moelle et de Thérapie Cellulaire (SFGM-TC) recently reported a registry study which analyzed 285 patients with PTCL who underwent alloSCT between 2006 and 2014 [53]. For the 138 patients who underwent frontline alloSCT in CR1 or PR1, the 4-year OS was 63%, the cumulative incidence of relapse was 19% at 2 years, and the TRM was 24% at 4 years.

### 2.3. What Can Be Learned from Studies Dedicated to A Single Nodal PTCL Entity?

The majority of studies on PTCLs have mixed the different histological entities, complicating the evaluation of prognostic factors and the impact of treatment for each PTCL subtype. A few studies, more often retrospective than prospective, have focused on a specific entity. These studies are presented in Table 6 [12,18,19,54,55,56,57,58,59,60,61,62].

Regarding AITL, a retrospective LYSA study which analyzed patients included in several clinical trials did not find any significant difference between CHOP and more intensive treatments such as doxorubicin, cyclophosphamide, vindesine, bleomycin, and prednisone (ACVBP) [54]. A Japanese study found similar survival (PFS and OS) with CHOP and pirarubicin, cyclophosphamide, vincristine, and prednisolone (THP-COP) regimens [55]. Two prospective phase 2 studies conducted by the LYSA did not show any benefit to the addition of rituximab or lenalidomide to CHOP compared to historical controls [56,58]. Finally, a study from the prospective International T-cell Project (ITCP) found that patients who underwent consolidative transplantation in CR1 had significantly better outcomes compared with transplant-eligible patients (age ≤ 65 years) who did not undergo transplantation, with 5-year PFS of 79% vs. 31%, and 5-year OS of 89% vs. 52%, respectively [59]. On the other hand, there was no significant difference in 5-year OS for patients receiving chemotherapy regimens with and without etoposide (50% vs. 43%, respectively).

Considering PTCL-NOS, the retrospective study from the IIL lymphoma registry did not find any benefit from bone marrow autologous transplantation [60]. In the retrospective study from the International Peripheral T-Cell Lymphoma (IPTCL) Project, there was no survival advantage for patients who received anthracycline-based combination chemotherapy compared with those receiving combination chemotherapy without anthracycline [61]. In the recent study from the prospective ITCP on ALK− ALCL, regimens containing both anthracycline and etoposide were associated with superior OS but not PFS [62].

Regarding ALK+ ALCL, a retrospective study from the Nordic Lymphoma Group and an individual-patient data pooled analysis from 6 studies (including 263 patients) support that the integration of etoposide into the CHOP (CHOEP) may be associated with important improvements in PFS and OS [12,19]. Of note, the benefit of CHOEP over CHOP was independent of IPI in both studies. Finally, in the pooled analysis, 34 patients underwent consolidative autoSCT (all were <60 years), and for patients <60 years in CR or PR, in stratified Cox models including etoposide-based induction, IPI and consolidative autoSCT, only the etoposide-based induction and the IPI remained independently prognostic for PFS and OS, without impact of autoSCT.

**Figure 2:**
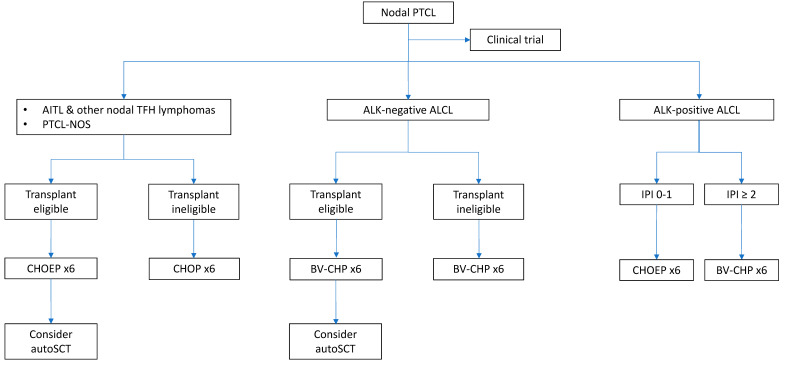
Proposed algorithm of frontline treatment in nodal PTCLs. This algorithm is based on histological subtype, transplant-eligibility, and prognostic factors.

A proposed treatment algorithm for newly diagnosed nodal PTCLs is shown in Figure 2.

## 3. Treatment of Relapsed/Refractory PTCLs

### 3.1. How Many Patients Relapse or Progress?

In the ITCP study, among 937 patients enrolled between 2006 and 2015 who received first-line treatment, 633 (68%) either progressed (47%) or relapsed (21%) [63]. Relapsed disease was defined as relapse at least 1 month from completion of front-line therapy in patients who achieved a CR or a satisfactory PR. Among the 197 relapsed patients, 125 (63%) had an early relapse (≤12 months), and 72 (37%) had a late relapse (>12 months).

In a retrospective cohort of 775 mainly nodal PTCL patients from the Mayo Clinic and the Swedish Lymphoma Registry treated with curative intent between 2000 and 2012, 582 (75%) had an event of relapse/progression, retreatment, or death from any cause [10]. A total of 64% of patients progressed/relapsed within the first 24 months.

### 3.2. What Is the Outcome after the First Event of Progression/Relapse?

The British Columbia Cancer Agency (BCCA) reported the survival of patients with relapsed/refractory (R-R) nodal PTCL diagnosed between 1976 and 2010, who were not transplanted after progression/relapse [64]. For the 153 analyzed patients (79 PTCL-NOS, 38 AITL, 25 ALK− ALCL (including 1 case with unknown ALK status), and 11 ALK+ ALCL), median time from initial diagnosis to progression/relapse after primary therapy was 6.7 months. After progression/relapse, median second PFS and OS were 3.1 and 5.5 months, respectively, without difference by PTCL subtype. Patients who received chemotherapy at relapse (*n* = 89) had a better median PFS and OS of 3.7 and 6.5 months, respectively, which was statistically significant, but clinically disappointing.

In the prospective ITCP study, the median OS of the 633 R-R patients was 5.8 months, with a 3-year OS of 23% [63]. The outcome was poorer for refractory versus relapsed patients, with median OS of 5 versus 11 months, and 3-year OS of 21% versus 28%, respectively (*p* < 0.001).

In the study from the Mayo Clinic and the Swedish Lymphoma Registry, patients who progressed/relapsed within the first 24 months had a median OS of 4.9 months and 5-year OS of 11% [10]. In contrast, median OS after achieving EFS24 was not reached, and 5-year OS was 78%.

The retrospective study from the Swedish Lymphoma Registry showed that central nervous system (CNS) involvement at progression/relapse in PTCLs occurred in 4.5% [65]. However, the poor outcome after relapse was largely driven by systemic rather than CNS disease and was not significantly altered by the presence of CNS involvement at relapse.

Overall, these studies showed that progressions/relapses of PTCLs are frequent (approximately 70% of patients), occur most often early (during the first year after initial diagnosis), and have a poor outcome, with a median OS of approximately 6 months. This survival rate did not significantly improve between 1976 and 2015. However, since that time, new treatments have improved results somewhat, at least in some subtypes, as we will see.

### 3.3. Second-Line Chemotherapy Regimen for PTCL-NOS and AITL

The same salvage combination regimens as for aggressive B lymphomas can be used, such as DHAP, DHAX (dexamethasone, cytarabine, oxaliplatin), ICE (ifosfamide, carboplatin, etoposide), GDP (gemcitabine, cisplatin, dexamethasone), ESHAP (etoposide, methylprednisolone, cytarabine, cisplatin), or GEMOX (gemcitabine, oxaliplatin) [66].

Single chemotherapy agents such as bendamustine or gemcitabine may be used. Bendamustine was assessed in the BENTLY phase 2 study [67]. For the 60 enrolled patients, the objective response rate (ORR) after 3 cycles was 50% (CR/CRu 28%), the median PFS and OS were 3.6 and 6.2 months, respectively. In a subsequent, French, real-life study on 138 R-R PTCLs, ORR was 33% (CR 25%), median PFS and OS were 3.1 and 4.4 months, respectively [68]. In a phase 2 study including 20 PTCL patients treated with gemcitabine, the ORR was 55% (CR 30%) [69]. Among the 6 CR patients, 5 were in continuous CR with a median duration of response of 34 months.

### 3.4. Treatment of R-R ALCL

In the pre-BV era, a study from the LYSA reported the outcome of ALK+ and ALK− ALCL after the first progression/relapse [70]. Among the 138 (64 ALK+ and 74 ALK− ALCL) adults initially treated in clinical trials, 40 (14 ALK+ and 26 ALK− ALCL) had a first progression or relapse. Most patients received standard salvage chemotherapy (mainly DHAP or ESHAP). The ORR to salvage chemotherapy was 54% (ALK+, 71%; ALK−, 47%; CR 46%, PR 8%). Median follow-up from the first R-R events was 12.5 years. For ALK+ and ALK− patients, respectively, median time between inclusion in first-line clinical trials and first R-R events was 6 and 11.1 months; median PFS after the first-R/R events were 3.8 and 5.3 months; median OS were 13.6 and 8.1 months, and 5-y OS were 36% and 19%, respectively.

A phase 2 study evaluated the safety and efficacy of BV in patients with R-R sALCL [71]. The primary endpoint was the ORR according to the 2007 revised response criteria. BV (1.8 mg/kg) was administered every 3 weeks for up to 16 cycles. Median observation time was 6 years from the start of treatment. Overall, 58 patients (16 ALK+ and 42 ALK− ALCL) were enrolled. A total of 50 patients achieved a best objective response (ORR 86%), including 38 (66%) CR and 12 (20%) PR. The median PFS was not reached for CR patients, 4.5 months for PR patients, 2.5 months for SD patients, and 1.1 for PD patients, meaning that CR should be the goal of BV treatment. Positron emission tomography (PET) after 4 cycles was highly predictive of outcome. In ALK+ and ALK− ALCL patients, 10/16 (63%) and 28/42 (67%) were in CR, respectively. Among the 10 ALK+ patients in CR, 6 underwent alloSCT, 1 underwent autoSCT, and 3 were not transplanted. At last follow-up, 4 alloSCT and 1 non-transplanted patients were still alive and in continuous CR. Among the 28 ALK− patients in CR, 2 underwent alloSCT, 7 underwent autoSCT, and 19 no transplantation. At last follow-up, 1 alloSCT, 5 autoSCT, and 11 non-transplanted patients were still alive and in continuous CR. For ALK+ and ALK− patients, the 5-year PFS rates were 37% and 39%, and the 5-year OS rates were 56% and 61%, respectively.

Overall, although this study was not randomized, its results showed better outcomes than historical controls in terms of response, PFS, and OS, and BV has become the standard treatment for R-R ALCL. Recently, a population-based study described the efficacy of BV monotherapy in R-R sALCL using Public Health England data [72]. For the 127 patients treated between 2014 and 2019, the 2-year OS was 47%, without significant difference according to ALK status. The majority of deaths occurred within 18 months, with very few events after this period.

For R-R ALK+ ALCL, some ALK inhibitors have been evaluated in children and adults. In adults, ALK inhibitors are mainly used after failure of BV treatment. Table 7 summarizes prospective studies [73,74,75,76,77,78].

### 3.5. What Is the Role of Hematopoietic Stem-Cell Transplantation in R-R PTCLs?

As previously shown, the BCCA reported the survival of patients with R-R nodal PTCLs who were not transplanted after progression/relapse [64]. Patients who received chemotherapy at relapse (*n* = 89) had a median PFS and OS of 3.7 and 6.5 months, respectively. The goal of transplantation is therefore to improve these results.

#### 3.5.1. Autologous Stem-Cell Transplantation

Studies on autoSCT in adults with R-R PTCLs are retrospective. A prospective pediatric study, the ALCL-relapse trial, assessed a risk-stratified treatment (alloSCT, autoSCT, or vinblastine monotherapy) of children with R-R ALK+ ALCL [79]. Table 8 summarizes studies that included at least 50 patients [63,80,81,82,83] and the pediatric ALCL-relapse trial. Overall, after autoSCT in CR/PR adults, the 3- to 5-y OS is approximately 50%.

#### 3.5.2. Allogeneic Stem-Cell Transplantation

There are only 1, small, prospective phase 2 study in adults, 1 prospective pediatric study in ALK+ ALCL, and retrospective studies mainly from registries [53,79,82,84,85,86,87]. Table 9 summarizes the prospective studies and the retrospective studies that included at least 50 patients.

Overall, after alloSCT, the 3 to 5-y OS is approximately 50%. Progressive disease at time of alloSCT is the dominant adverse factor. However, even in this situation, prolonged survival is observed in about 35% of cases. An altered performance status is the second factor associated with a reduced OS. Finally, in recent years, the non-relapse mortality (NRM) has improved and is now approximately 20% at 3 years.

#### 3.5.3. Autologous or Allogeneic Stem-Cell Transplantation?

Based on non-randomized studies, autoSCT and alloSCT in R-R PTCLs improved the outcome compared to no transplantation. The question is whether it is better to perform an autoSCT or an alloSCT. In the absence of a randomized study, this question cannot be formally answered. Recently, a systematic review and meta-analysis compared the effectiveness and safety of autoSCT vs. alloSCT in patients with R-R PTCLs [88]. A total of 30 studies (including 885 patients who underwent autoSCT and 880 who underwent alloSCT) were analyzed. In the autoSCT group, 5-year PFS and OS rates were 40% and 53%, respectively, and the 3-year TRM was 7%. In the alloSCT group, 5-year PFS and OS rates were 48% and 54%, respectively, and the 3-year TRM was 32%. The conclusion was that PFS and OS were similar in the autoSCT and alloSCT groups; however, alloSCT was associated with specific survival benefits.

Concerning ALK+ ALCL, the prospective pediatric ALCL-relapse trial assessed a risk-stratified treatment (alloSCT, autoSCT, or vinblastine monotherapy) of children with R-R ALK+ ALCL [79]. AlloSCT was highly efficacious for patients with a high-risk early relapse (within the first year after initial diagnosis) or refractory disease, whereas autoSCT did not prevent additional relapse in early relapsed ALCL. The main conclusion of the study was that autoSCT is not indicated for R-R ALK+ ALCL. Except for low-risk patients (with a late relapse) for whom vinblastine monotherapy is an efficacious treatment option, all other R-R patients should be referred for alloSCT. In the absence of prospective data in adults, this approach could be applied in adults with R-R ALK+ ALCL.

### 3.6. Novel Agents

Several novel agents have been assessed in R-R PTCLs, and, while some have received approval for clinical use, approval is not uniform across countries. BV and ALK inhibitors have been previously described. Table 10 and Table 11 summarize the prospective studies of other single agents [89,90,91,92,93,94,95,96,97,98,99,100,101,102,103,104,105,106,107,108] and combinations [109,110,111,112,113,114,115,116,117], respectively.

Overall, with single agents, ORR ranged from 0 to 50%, CR rates from 0 to 27%, and median PFS was most often less than 6 months. Combination studies included small numbers of patients, making it difficult to interpret the results on both efficacy and toxicity. Larger studies are therefore necessary.

A study from the COMPLETE registry compared treatment results for patients with R-R PTCLs who received single agents to those who received combination chemotherapy [118]. Single agents were as follows: romidepsin, belinostat, BV, alisertib, lenalidomide, denileukin diftitox, bendamustine, or pralatrexate. Combination included any multiagent chemotherapy regimen excluding the above single agents. At first retreatment, 31 patients received single agents and 26 received combination therapy. The CR rate was higher with single agents vs. combination therapy (41% vs. 19%; *p* = 0.02). After a median follow-up of 2 years, median PFS (11.2 vs. 6.7 months; *p* = 0.02), and OS (38.9 vs. 17.1 months; *p* = 0.02) were longer with single agents. This study suggests better results with single agent treatment, but in the absence of randomized study, the debate remains open.

## 4. Conclusions

The management of PTCL patients continues to be a challenge for physicians, and its treatment is still an unmet medical need. The historical “one-size-fits-all” PTCL treatment approach has been shown to be ineffective and should no longer be used. Recent advances in the biology of PTCLs have led to the identification of new targets, and to the establishment of biomarker-driven treatments such as BV for ALCL and ALK inhibitors for ALK+ ALCL. Given the differences between PTCL entities in terms of biology, lymphomagenesis, response to treatment, and outcome, future clinical trials should be oriented primarily by PTCL entity, and possibly by a common molecular target. This will require multicentric collaboration to facilitate participation in clinical trials.

## Figures and Tables

**Table 1:** Published studies of frontline CHOEP in PTCLs.

Study Type	PTCL Subtype	*N*	Main Results	Reference
Retrospective (Patients treated within trials of the DSHNHL)	Nodal and extranodal	320 (subgroup analysis)	(1) In pts < 60 years with normal LDH, CHOEP (*n* = 42) improved 3-year EFS over CHOP (*n* = 41): 75% vs. 51% (*p* = 0.003); OS not affected. (2) ALK+ ALCL < 60 years with normal LDH: CHOEP (*n* = 34) improved 3-year EFS over CHOP (*n* = 12): 91% vs. 57% (*p* = 0.012).	Schmitz N, 2010 [7]
Phase 2 (NLG-T-01)	Nodal and extranodal (ALK+ ALCL excluded)	160	CHOEP-14 x6 (etoposide omitted in pts > 60 years) followed by BEAM-autoSCT: 5-y PFS = 44%, 5-y OS = 51%.	d’Amore F, 2012 [14]
Retrospective (Swedish Lymphoma Registry)	Nodal and extranodal	755 (subgroup analysis)	In pts ≤ 60 years with ALK− ALCL, AITL, PTCL NOS, EATL, and TCL-U, CHOEP improved PFS over CHOP (HR 0.49; *p* = 0.008) in multivariate analysis; OS not affected.	Ellin F, 2014 [9]
Retrospective (Korean National Health Insurance Service and National Cancer Registry)	Nodal and extranodal (ENKTCL excluded)	1933 (subgroup analysis)	CHOEP did not improve PFS and OS over CHOP.	Kim YA, 2017 [15]
Retrospective (Czech Lymphoma Study Group database)	Nodal and extranodal PTCL, CTCL, T-ALL/LBL	906 (subgroup analysis)	ALK+ ALCL excluded for the comparison of CHOEP and CHOP. CHOEP (*n* = 68) improved 5-y PFS (59% vs. 33%, *p* = 0.001) and OS (66% vs. 48%, *p* = 0.008) over CHOP (*n* = 113), in multivariate analysis.	Janikova A, 2019 [16]
Randomized, phase 3 (AATT; autoSCT vs. alloSCT)	Nodal and extranodal (ALK+ ALCL excluded)	104	CHOEP-14 x4 followed by DHAP x1 followed by autoSCT or alloSCT. No significant difference between autoSCT and alloSCT arms: 3-y EFS 38% vs. 43% and 3-y OS 70% vs. 57%, respectively.	Schmitz N, 2021 [17]
Prospective (International T-cell Project)	ALK− ALCL	235 (subgroup analysis)	Treatment with etoposide (*n* = 31) improved 5-y OS (69% vs. 44%, *p* = 0.05) over treatment without etoposide (*n* = 168); PFS not affected.	Shustov A, 2021 [18]
Retrospective (Nordic Lymphoma Group)	ALK+ ALCL	122 (subgroup analysis)	In pts 41–65 years CHOEP (*n* = 27) improved 5-y OS over CHOP (*n* = 17): 78% vs. 47% (HR = 0.38, *p*= 0.047).	Cederleuf H, 2017 [19]
Retrospective (pooled analysis of individual patient data from 6 studies)	ALK+ ALCL	263 (subgroup analysis)	CHOEP (*n* = 38) improved 5-y PFS (89% vs. 57%, *p* = 0.002) and OS (97% vs. 69%, *p* = 0.001) over CHOP (*n* = 98).	Sibon D, 2019 [12]

Abbreviations. AITL, angioimmunoblastic T-cell lymphoma; ALCL, anaplastic large cell lymphoma; ALK, anaplastic lymphoma kinase; alloSCT, allogeneic stem cell transplantation; autoSCT, autologous stem cell transplantation; BEAM, carmustine, etoposide, cytarabine, and melphalan; CHOP, cyclophosphamide, doxorubicin, vincristine, and prednisone; CHOEP, CHOP + etoposide; CTCL, cutaneous T-cell lymphoma; DHAP, cisplatin, cytarabine, and dexamethasone; DSHNHL, German High-Grade Non-Hodgkin Lymphoma Study Group; EATL, enteropathy-associated T-cell lymphoma; EFS, event-free survival; ENKTCL, extranodal NK/T-cell lymphoma; LDH, lactate dehydrogenase; LYSA, The Lymphoma Study Association; OS, overall survival; PFS, progression-free survival; PTCL, peripheral T-cell lymphoma; PTCL-NOS, PTCL not otherwise specified; SCT, stem-cell transplantation; T-ALL/LBL, T-acute lymphoblastic leukemia/lymphoblastic lymphoma; TCL-U, T-cell lymphoma, unspecified; THP-COP, pirarubicin, cyclophosphamide, vincristine, and prednisolone.

**Table 2:** Phase 1 and 2 studies combining CHOP/CHOP-like with novel agents (published studies).

Regimen	Phase	*n*	ORR/CR	Survival	Reference
Alemtuzumab-CHOP	2	24	75%/71%	3-y FFS: 48% 3-y OS: 53%	Gallamini A, 2007 [25]
Alemtuzumab-DA-EPOCH	1–2	30	83%/57%	2-y PFS: 32% 2-y OS: 49%	Roswarski J, 2019 [26]
Bortezomib-CHOP	2	46	76%/65%	3-y PFS: 35% 3-y OS: 47%	Kim SJ, 2012 [27]
Denileukin diftitox-CHOP	2	49	65%/55%	2-y PFS: 43% 2-y OS: 65%	Foss FM, 2013 [28]
Vorinostat-CHOP	1	14	86%/86%	2-y PFS: 79% 2-y OS: 81%	Oki Y, 2013 [29]
Romidepsin-CHOP	1b-2	37	68%/51%	2.5-y PFS: 41% 2.5-y OS: 71%	Dupuis J, 2015 [30]
Everolimus-CHOP	2	30	90%/57%	2-y PFS: 33% 2-y OS: 70%	Kim SJ, 2016 [31]
Pralatrexate-CEOP	2	33	70%/52%	2-y PFS: 39% 2-y OS: 60%	Advani RH, 2016 [32]
BV-CHP	1	26 including 19 ALCL (3 ALK+; 16 ALK−)	100%/92%	5-y PFS: 52% (ALK− 38%) 5-y OS: 80% (ALK− 75%)	Fanale MA, 2018 [33]
Belinostat-CHOP	1	23	86%/71%	-	Johnston PB, 2021 [34]

Abbreviations. ALCL, anaplastic large cell lymphoma; ALK, anaplastic lymphoma kinase; BV-CHP, brentuximab vedotin, cyclophosphamide, doxorubicin, and prednisone; CEOP, cyclophosphamide, etoposide, vincristine, and prednisone; CHOP, cyclophosphamide, doxorubicin, vincristine, and prednisone; CR, complete response; DA-EPOCH, dose-adjusted etoposide, prednisone, vincristine, cyclophosphamide, and doxorubicin; FFS, failure-free survival; ORR, objective response rate; OS, overall survival; PFS, progression-free survival.

**Table 3:** Randomized (published) studies in frontline treatment of PTCLs.

Study	Regimen Compared with CHOP	Primary Endpoint	*n*	ORR/CR	Survival	Reference
LTP-95	VIP-rABVD	EFS	VIP-rABVD (43) CHOP (45)	58%/44% 67%/33%	2-y EFS: 45% (VIP-rABVD) vs. 41% (CHOP) 2-y OS: 55% (VIP-rABVD) vs. 55% (CHOP)	Simon A, 2010 [36]
CHEMO-T	GEM-P	CR/CRu	GEM-P (44) CHOP (43)	68%/46% 76%/62%	2-y PFS: 38% (GEM-P) vs. 37% (CHOP) 2-y OS: 64% (GEM-P) vs. 51% (CHOP)	Gleeson M, 2018 [11]
ECHELON-2	BV-CHP	Modified PFS by independent central review	BV-CHP (226) CHOP (226)	83%/68% 72%/56%	3-year PFS 57% (BV-CHP) vs. 44% (CHOP) 3-year OS 77% (BV-CHP) vs. 69% (CHOP)	Horwitz S, 2019 [37]
ECHELON-2 (updated)	BV-CHP	Modified PFS by independent central review	BV-CHP (226) CHOP (226)	-	5-year PFS 51% (BV-CHP) vs. 43% (CHOP) 5-year OS 70% (BV-CHP) vs. 61% (CHOP)	Horwitz S, 2022 [38]
ACT-2	Alemtuzumab-CHOP	EFS	A-CHOP (58) CHOP (58)	72%/60% 66%/43%	3-y EFS: 27% (A-CHOP) vs. 24% (CHOP) 3-y OS: 37% (A-CHOP) vs. 56% (CHOP)	Wulf GG, 2020 [39]
Ro-CHOP	Romidepsin-CHOP	PFS	Ro-CHOP (211) CHOP (210)	63%/41% 60%/37%	2-y PFS: 43% (Ro-CHOP) vs. 36% (CHOP) 2-y OS: 64% (Ro-CHOP) vs. 63% (CHOP)	Bachy E, 2022 [13]

Abbreviations. BV-CHP, brentuximab vedotin, cyclophosphamide, doxorubicin, and prednisone; CHOP, cyclophosphamide, doxorubicin, vincristine, and prednisone; CR, complete response; CRu, CR unconfirmed; EFS, event-free survival; GEM-P, gemcitabine, cisplatin, and methylprednisolone; ORR, objective response rate; OS, overall survival; PFS, progression-free survival.

**Table 4:** Prospective, published, phase 2 studies of consolidative autoSCT in PTCL.

Reference	PTCL Subtype	*n*	Induction Regimen	ORR/CR Prior to autoSCT	Transplantation Rate	Survival	TRM
Corradini P, 2006 [44]	All subtypes including 30% ALK+ ALCL	62	Intensified regimen or MACOP-B	72%/56%	74%	12-y EFS: 30% 12-y OS: 34%	4.8%
Rodriguez J, 2007 [45]	Nodal PTCL excluding ALK+ ALCL	26	MegaCHOP	73%/46%	73%	3-y PFS 53% 3-y OS 73%	0%
Mercadal S, 2008 [46]	All subtypes including 1 ALK+ ALCL	41	High-dose CHOP/ESHAP	59%/49%	41%	4-y PFS: 30% 4-y OS: 39%	0%
Reimer P, 2009 [47]	All subtypes excluding ALK+ ALCL	83	CHOP	79%/39%	66%	3-y PFS: 36% 3-y OS: 48%	3.6%
D’Amore F, 2012 [14]	All subtypes excluding ALK+ ALCL	160	CHOEP	82%/51%	72%	5-y PFS: 44% 5-y OS: 51%	4%
Wilhelm M, 2016 (extension and update of Reimer P, 2009) [47,48]	All subtypes excluding ALK+ ALCL	111	CHOP	82%/62%	68%	5-y PFS: 39% 5-y OS: 44%	3.6%

Abbreviations. ALCL, anaplastic large cell lymphoma; ALK, anaplastic lymphoma kinase; autoSCT, autologous stem-cell transplantation; CHOP, cyclophosphamide, doxorubicin, vincristine, and prednisone; CHOEP, CHOP + etoposide; CR, complete response; EFS, event-free survival; ESHAP, etoposide, methylprednisolone, cytarabine, cisplatin; MACOP-B, methotrexate, doxorubicin, cyclophosphamide, vincristine, prednisone, and bleomycin; ORR, objective response rate; OS, overall survival; PFS, progression-free survival; PTCL, peripheral T-cell lymphoma; TRM, transplant-related mortality.

**Table 5:** Selected, published retrospective studies of consolidative autoSCT in PTCLs, including at least 100 patients.

Reference	Type of Study	PTCL Subtype	Main Results
Ellin F, 2014 [9]	Swedish Lymphoma Registry	Nodal PTCL and EATL excluding ALK+ ALCL	In an intention-to-treat analysis in 252 nodal PTCLs and EATL patients excluding ALK+ ALCL, planned upfront autoSCT (*n* = 128) was associated with a superior PFS (HR, 0.56; *p* = 0.002) and OS (HR, 0.58; *p* = 0.004) compared with patients treated without autoSCT (*n* = 124).
Fossard G, 2018 [51]	LYSA study	Nodal PTCL excluding ALK+ ALCL	In an intention-to-treat analysis in 269 nodal PTCL patients excluding ALK+ ALCL, there was no significant difference in PFS and OS between planned upfront autoSCT group (*n* = 134) and patients treated without autoSCT (*n* = 135).
Sibon D, 2019 [12]	International pooled analysis	ALK+ ALCL	Among 263 pts, 34 underwent upfront autoSCT (all were <60 years). For patients <60 years in CR or PR, in stratified Cox models including etoposide-based induction, IPI and upfront autoSCT, only the etoposide-based induction and the IPI remained independently prognostic for PFS and OS, without impact of autoSCT

Abbreviations. ALCL, anaplastic large cell lymphoma; ALK, anaplastic lymphoma kinase; autoSCT, autologous stem-cell transplantation; CR, complete response; EATL, enteropathy-associated T-cell lymphoma; HR, hazard ratio; IPI, international prognostic index; OS, overall survival; PFS, progression-free survival; PR, partial response; PTCL, peripheral T-cell lymphoma.

**Table 6:** Published studies focused on a single nodal PTCL entity.

PTCL Subtype	Study Type	Treatment	*n*	CR Rate	Survival	Reference
AITL	Retrospective (LYSA)	Mainly CHOP and ACVBP	157	46%	2-y EFS: 38% 2-y OS: 51%	Mourad N, 2008 [54]
AITL	Retrospective (Japan)	Mainly CHOP and THP-COP	207	66%	3-y PFS: 38% 3-y OS: 54%	Tokunaga T, 2012 [55]
AITL	Phase 2 (LYSA)	Rituximab-CHOP	25	44%	2-y PFS: 42% 2-y OS: 62%	Delfay-Larue MH, 2012 [56]
AITL	Retrospective (IPTCL)	Mainly CHOP	243	61%	5-y FFS: 18% 5-y OS: 32%	Federico M, 2013 [57]
AITL	Phase 2 (LYSA)	Lenalidomide-CHOP	78	41%	2-y PFS: 42% 2-y OS: 59%	Lemonnier F, 2021 [58]
AITL	Prospective observational (ITCP)	Mainly CHOP	282	51%	3-y PFS: 38% 3-y OS: 50%	Advani RH, 2021 [59]
PTCL-NOS	Retrospective (IIL Lymphoma Registry)	Mainly CHOP	385	53%	5-y OS: 43%	Gallamini A, 2004 [60]
PTCL-NOS	Retrospective (IPTCL)	Mainly CHOP	340	56%	5-y FFS: 20% 5-y OS: 32%	Weisenburger DD, 2011 [61]
PTCL-NOS	Prospective observational (ITCP)	Mainly CHOP	311	ND	5-y PFS: 23% 5-y OS: 32%	Federico M, 2018 [62]
ALK− ALCL	Prospective observational (ITCP)	Mainly CHOP	235	63%	5-y PFS: 43% 5-y OS: 49%	Shustov A, 2021 [19]
ALK+ ALCL	Retrospective (Nordic Lymphoma Group)	CHOEP and CHOP	CHOEP *n* = 27 CHOP *n* = 17 (pts 41–65 years)	ND	5-y OS CHOEP: 78% CHOP: 47%	Cederleuf H, 2017 [18]
ALK+ ALCL	Retrospective (international pooled analysis)	Mainly CHOEP and CHOP	CHOEP *n* = 38 CHOP *n* = 98 (pts ≥ 18 years)	ND	5-y OS CHOEP: 97% CHOP: 69%	Sibon D, 2019 [12]

Abbreviations. ACVBP, doxorubicin, cyclophosphamide, vindesine, bleomycin, prednisone; AITL, angioimmunoblastic T-cell lymphoma; ALCL, anaplastic large cell lymphoma; ALK, anaplastic lymphoma kinase; CHOP, Cyclophosphamide, doxorubicin, vincristine, and prednisone; CHOEP, CHOP + etoposide; CR, complete response; EFS, event-free survival; FFS, failure-free survival; IIL, Intergruppo Italiano Linfomi; IPTCL, International Peripheral T-Cell Lymphoma Project; ITCP, International T-cell Project; LYSA, The Lymphoma Study Association; OS, overall survival; PFS, progression-free survival; PTCL, peripheral T-cell lymphoma; PTCL-NOS, PTCL not otherwise specified; THP-COP, pirarubicin, cyclophosphamide, vincristine, and prednisolone.

**Table 7:** Prospective studies on ALK inhibitors for R-R ALK+ ALCL.

Reference	ALK Inhibitor	Phase	*n* and Dosage	Median Age	ORR/CR	PFS
Brugières L, 2017 [73]	Crizotinib	2	*n* = 18 250 mg ×2/d (adults) 165 mg/m^2^ ×2/d (children)	20 (1–60)	67%/40%	1-y PFS 48%
Mossé Y, 2017 [74]	Crizotinib	1–2	*n* = 6 (165 mg/m^2^ ×2/d) *n* = 20 (280 mg/m^2^ ×2/d)	5.9 (3–13) 12.2 (6–20)	83%/83% 90%/80%	-
Gambacorti-Passerini C, 2018 [75]	Crizotinib	1b	*n* = 18 (250 mg ×2/d)	25 (15–37)	53%/47%	2-y PFS 63%
Bossi E, 2020 [76]	Crizotinib	2	*n* = 12 (250 mg ×2/d)	31 (18–83)	83%/58%	2-y PFS 65%
Fukano R, 2020 [77]	Alectinib	2	*n* = 10 300 mg ×2/d (≥35 kg) 150 mg ×2/d (<35 kg)	19.5 (6–70)	80%/60%	1-y PFS 58%
Fischer M, 2021 [78]	Ceritinib	1	*n* = 8 (500 mg/m^2^)	Children	75%/25%	1-y PFS 67%

Abbreviations. CR, complete response; ORR, objective response rate; PFS, progression-free survival.

**Table 8:** Published retrospective studies of autoSCT in R-R PTCLs (of at least 50 patients).

Study	PTCL Subtype	*n*	Survival	TRM
Rodriguez J, 2003 [80]	All subtypes	78	5-y OS: 45%	8%
Nademanee A, 2011 [81]	All subtypes	55	5-y OS: 45%	0%
Smith S, 2013 [82]	All subtypes	94	3-y OS: 53%	6%
El-Asmar J, 2016 (Meta-analysis) [83]	All subtypes	581	OS 47%	10%
Bellei M, 2018 [63]	All subtypes	99 (compared to 124 eligible CR-PR patients, not transplanted)	Transplanted: 3-y OS: 48% Not transplanted: 3-y OS: 27%	-
Knörr F, 2020 (prospective) [79]	ALK+ ALCL (≤21 years)	22	5-y EFS: 41% 5-y OS: 82%	2 deaths

Abbreviations. CR, complete response; OS, overall survival; PR, partial response; PTCL, peripheral T-cell lymphoma; TRM, transplant-related mortality.

**Table 9:** Published studies of alloSCT in R-R PTCLs (of at least 50 patients for retrospective studies).

Study	PTCL Subtype	*N*	Survival	NRM
Corradini P, 2004 (prospective) [84]	Nodal PTCL excluding ALK+ ALCL	17	3-y PFS: 64% 3-y OS: 81%	6% at 2 years
Knörr F, 2020 (prospective) [79]	ALK+ ALCL (≤21 years)	36	5-y EFS: 81% 5-y OS: 83%	2 deaths
Le Gouill S, 2008 [85]	All subtypes	77	5-y EFS: 53% 5-y OS: 57%	33% at 5 years
Dodero A, 2012 [86]	All subtypes	52	5-y PFS: 40% 5-y OS: 50%	12% at 5 years
Smith S, 2013 [82]	All subtypes	93	Myeloablative: 3-y PFS: 29% 3-y OS: 31% Non-myeloablative: 3-y PFS: 32% 3-y OS: 50%	Myeloablative: 34% at 3 years Non myeloablative: 27% at 3 years
Epperla N, 2019 [87]	AITL	249	4-y PFS: 49% 4-y OS: 56%	30% at 4 years
Mamez AC, 2020 [53]	All subtypes	147	CR ≥ 2 or PR ≥ 2: 4-y OS: 61% Progressive disease: 4-y OS: 37%	CR ≥ 2 or PR ≥ 2: 30% at 4 years Progressive disease: 40% at 4 years

Abbreviations. AITL, angioimmunoblastic T-cell lymphoma; ALCL, anaplastic large cell lymphoma; ALK, anaplastic lymphoma kinase; CR, complete response; EFS, event-free survival; OS, overall survival; PFS, progression-free survival; PR, partial response; PTCL, peripheral T-cell lymphoma; NRM, non-relapse mortality.

**Table 10:** Published, prospective studies of novel agents in R-R PTCLs (single agent studies).

Agent	Target	Phase	No. of Evaluable Patients (Excluding CTCL)	ORR/CR	Survival	Reference
Romidepsin	HDAC	2	130	25%/15%	Median PFS 4 mo	Coiffier B, 2012 [89]
Belinostat	HDAC	2	120	26%/11%	Median PFS 1.6 mo Median OS 7.9 mo	O’Connor OA, 2015 [90]
Chidamide	HDAC	2	79	28%/14%	Median PFS 2.1 mo Median OS 21.4 mo	Shi Y, 2015 [91]
Alisertib	Aurora A kinase	2	30	30%/7%	1-y PFS: 8%; Median 3 mo 1-y OS: 30%; Median 8 mo	Barr PM, 2015 [92]
Alisertib	Aurora A kinase	3	271	33%/18%	Median PFS 115 versus 104 days for the comparator arm	O’Connor OA, 2019 [93]
Copanlisib	PI3K-α/δ	2	14	21%/14%	-	Dreyling M, 2017 [94]
Duvelisib	PI3K-δ/γ	1	16	50%/19%	Median PFS 8.3 mo Median OS 8.4 mo	Horwitz SM, 2018 [95]
Tenalisib	PI3K-δ/γ	1	15	47%/20%	-	Huen A, 2020 [96]
Ibrutinib	BTK	1	7	0%/0%	-	Kumar A, 2018 [97]
Ruxolitinib	JAK1/2	2	45	27%/7%	Median PFS * 2.8 mo Median OS * 26.2 mo	Moskowitz AJ, 2021 [98]
Dasatinib	Multiple kinases	1	9	29%/22%	Median PFS 2.5 mo Median OS 4.5 mo	Umakanthan JM, 2019 [99]
Lenalidomide	Immunomodulation Angiogenesis	2	54	22%/11%	Median PFS 2.5 mo	Morschhauser F, 2013 [100]
Lenalidomide	Immunomodulation Angiogenesis	2	39 (8 in first-line)	26%/8%	Median PFS 4 mo Median OS 12 mo	Toumishey E, 2015 [101]
Pembrolizumab	PD1	2	15	33%/27%	Median PFS 3.2 mo Median OS 10.6 mo	Barta SK, 2019 [102]
Geptanolimab	PD1	2	89	40%/15%	Median PFS 2.7 mo Median OS 14.6 mo	Shi Y, 2021 [103]
Plitidepsin	eEF1A2	2	29	21%/7%	Median PFS 1.6 mo Median OS 10.2 mo	Ribrag V, 2013 [104]; Losada A [105]
Pralatrexate	Folates	2	111	29%/11%	Median PFS 3.5 mo Median OS 14.5 mo	O’Connor OA, 2011 [106]
Ixazomib	Proteasome	2	7	14%/14%	-	Boonstra PS, 2017 [107]
Carfilzomib	Proteasome	1	12	25%/17%	Median PFS 2.8 mo Median OS 23 mo	Krishnan M, 2022 [108]

Abbreviations. BTK, Bruton tyrosine kinase; CR, complete response; CTCL, cutaneous T-cell lymphoma; eEFIA2, eukaryotic translation elongation factor 1 alpha 2; HDAC, histone deacetylase; JAK, Janus kinase; ORR, objective response rate; OS, overall survival; PD1, programmed cell death protein 1; PFS, progression-free survival; PI3K, phosphatidylinositol 3-kinase; PTCL, peripheral T-cell lymphoma. * Median PFS and OS of 52 patients (45 PTCLs + 7 mycosis fungoides).

**Table 11:** Published, prospective studies of novel agents in R-R PTCLs (combination studies).

Agent	Phase	No. of Evaluable Patients (Excluding CTCL)	ORR/CR	Survival	Reference
Lenalidomide-Vorinostat—Dexamethasone	1–2	8	25%/12.5%	Median PFS 2.2 mo Median OS 6.7 mo	Hopfinger G, 2014 [109]
Panobinostat—Bortezomib	2	23	43%/22%	Median PFS 2.6 mo Median OS 9.9 mo	Tan D, 2015 [110]
Gemcitabine—Romidepsin	2	20	30%/15%	Median PFS 2.5 mo Median OS 22 mo	Pellegrini C, 2016 [111]
Bortezomib—Romidepsin	1	2	0%/0%	-	Holkova B, 2017 [112]
Romidepsin—Pralatrexate	1	14	71%/29%	Median PFS 4.4 mo Median OS 12.4 mo	Amengual J, 2018 [113]
Romidepsin—ICE	1	15	93%/80%	Median PFS 10 mo Median OS 15 mo	Strati P, 2018 [114]
Oral 5-Azacytidine—Romidepsin	1	6	71%/71%	Median PFS not reached after a median follow-up of 15.3 mo	O’Connor OA, 2019 [115]
Selinexor—DICE	1	10	91%/82%	Median OS not reached after a median follow-up of 32.3 mo	Tang T, 2020 [116]
Gemcitabine—Copanlisib	1–2	25 (phase 2)	72%/32%	Median PFS 6.9 mo Median OS not reached	Yhim H-Y, 2021 [117]

Abbreviations. CR, complete response; CTCL, cutaneous T-cell lymphoma; mo, months; DICE, dexamethasone, ifosfamide, carboplatin, etoposide; ICE, ifosfamide, carboplatin, etoposide; ORR, objective response rate; OS, overall survival; PFS, progression-free survival; PTCL, peripheral T-cell lymphoma.
